# Wearing the Future—Wearables to Empower Users to Take Greater Responsibility for Their Health and Care: Scoping Review

**DOI:** 10.2196/35684

**Published:** 2022-07-13

**Authors:** Harjeevan Singh Kang, Mark Exworthy

**Affiliations:** 1 College of Medical and Dental Sciences University of Birmingham Birmingham United Kingdom; 2 Health Services Management Centre University of Birmingham Birmingham United Kingdom

**Keywords:** wearable, device, tracker, activity tracker, fitness tracker, technology, MedTech, HealthTech, sensor, monitor, gadget, smartwatch, empowerment, self-care, management, behavior, responsibility, attitude, personalization, mobile phone, self-management, smartphone, wearable electronic devices, health promotion, health behavior, mHealth, digital health, health care wearables, scoping review

## Abstract

**Background:**

Wearables refer to devices that are worn by individuals. In the health care field, wearables may assist with individual monitoring and diagnosis. In fact, the potential for wearable technology to assist with health care has received recognition from health systems around the world, including a place in the strategic Long Term Plan shared by the National Health Service in England. However, wearables are not limited to specialist medical devices used by patients. Leading technology companies, including Apple, have been exploring the capabilities of wearable health technology for health-conscious consumers. Despite advancements in wearable health technology, research is yet to be conducted on wearables and empowerment.

**Objective:**

This study aimed to identify, summarize, and synthesize knowledge on how wearable health technology can empower individuals to take greater responsibility for their health and care.

**Methods:**

This study was a scoping review with thematic analysis and narrative synthesis. Relevant guidance, such as the Arksey and O’Malley framework, was followed. In addition to searching gray literature, we searched MEDLINE, EMBASE, PsycINFO, HMIC, and Cochrane Library. Studies were included based on the following selection criteria: publication in English, publication in Europe or the United States, focus on wearables, relevance to the research, and the availability of the full text.

**Results:**

After identifying 1585 unique records and excluding papers based on the selection criteria, 20 studies were included in the review. On analysis of these 20 studies, 3 main themes emerged: the potential barriers to using wearables, the role of providers and the benefits to providers from promoting the use of wearables, and how wearables can drive behavior change.

**Conclusions:**

Considerable literature findings suggest that wearables can empower individuals by assisting with diagnosis, behavior change, and self-monitoring. However, greater adoption of wearables and engagement with wearable devices depend on various factors, including promotion and support from providers to encourage uptake; increased short-term investment to upskill staff, especially in the area of data analysis; and overcoming the barriers to use, particularly by improving device accuracy. Acting on these suggestions will require investment and constructive input from key stakeholders, namely users, health care professionals, and designers of the technology. As advancements in technology to make wearables viable health care devices have only come about recently, further studies will be important for measuring the effectiveness of wearables in empowering individuals. The investigation of user outcomes through large-scale studies would also be beneficial. Nevertheless, a significant challenge will be in the publication of research to keep pace with rapid developments related to wearable health technology.

## Introduction

### Background

#### Wearable Health Technology

Wearables are “seamlessly embedded portable computers...worn on the body” [1]. Examples include consumer products marketed as wellness gadgets, such as smartwatches produced by Apple [2] or activity trackers from Fitbit [3], and more specialized medical devices, such as those that can detect electrolyte levels [4] or screen blood for cancer cells [5].

Wearable devices can be used in the medical field to monitor individuals and assist with diagnosis, thereby enabling individuals to contribute to their health [6] and gain greater control of their lives [7]. For example, certain wearables have been developed to recognize the symptoms of COVID-19 infection by measuring individuals’ vital signs [8].

As technology advances, it may be expected that wearables will become more advanced in their health care capabilities. A future vision for wearables has been discussed [9], concerning the potential application of on-teeth sensors, smart contact lenses, electronic epidermal tattoos, smart patches, and smart textiles. Any data from wearables may be integrated with health systems and potentially inform care plans.

#### Empowerment

Patient empowerment has been well discussed in the literature, but the complexity of the concept is thought to be responsible for the “lack of a consensus definition” [10]. The most commonly cited definitions [11,12] indicate that “Patient empowerment starts from the principle of one’s inherent capacity to be responsible for one’s own life, and can be described as a complex experience of personal change, possibly facilitated by health care providers” [10]. Other researchers have proposed that patient empowerment encompasses activities that foster self-management [13].

Participatory health informatics (PHI) considers the role of technology in assisting individuals with self-management and decision-making by also improving health literacy and the physician-patient relationship so that individuals can become more involved in the aspects of their health and care [14]. Historically, research in the PHI field has predominantly been based on social media and internet-based applications, with patient empowerment having been identified as the most common theme in this body of research [14]. However, wearables are just beginning to be considered as part of PHI given recent technological advancements [14]. Therefore, similar research is now required to examine whether wearables can empower individuals in ways similar to those mentioned earlier regarding domains such as self-management, decision-making, and the physician-patient relationship.

There are several ways in which wearables may assist in empowering patients. First, wearables may minimize the impact of health care on the daily routine of patients. Wearables may offer greater convenience [15] if they reduce the need for patients to invest time in booking appointments with health care professionals, plan their schedule around such appointments, or commit time and money for appointment-related travel. Wearables have already been shown to reduce the need for certain in-person appointments [16].

Next, wearables collecting data throughout the day may provide a richer data set [17] than snapshot reading records obtained during visits to a health care facility. Such data may be collected more readily around individuals’ normal daily activities, whether at rest or on exertion [18], which may be useful for heart rate readings, for example.

Furthermore, patients can take an electrocardiogram (ECG) and other readings multiple times each day over the course of months. This would add to the richness of the data set and potentially better inform diagnosis and treatment while also proving valuable in screening for COVID-19 infection, as Apple Watch could regularly monitor blood oxygen levels [19]. Attending appointments for taking such readings would neither allow the degree of frequency nor convenience of doing so at home and while on the move as with wearables.

Moreover, wearables may help preserve patient dignity when offering an alternative to more privacy-intrusive procedures. For example, an ECG taken by Apple Watch [19] may be preferred over a traditional ECG in a medical setting, which would require the removal of clothing to expose the patient’s chest. Data from wearables may also flag early warning signs [2], prompting individuals to arrange appropriate medical consultations.

In addition, wearables may facilitate behavior change and potentially motivate patients to exercise, whether through daily step challenges, goal setting, or otherwise [20]. This could deliver associated health benefits [21] and help combat the obesity epidemic that faces health systems [22] and has been worsened by the COVID-19 pandemic [23].

#### Benefits for the Health System

The COVID-19 pandemic has exacerbated the pressure on the National Health Service (NHS) in England, as disruption to services has contributed to a backlog of care that is estimated to cost the NHS £2 billion (US $2.44 billion) to clear [24]. The NHS has been persistently overstretched, such that these additional pressures compound pre-existing problems of inadequate funding and understaffing [25]. As the NHS continues to face challenges, owing to resource constraints, care must be delivered more efficiently.

Innovative solutions are known to secure growth [26] by redefining care pathways [27] to improve patient satisfaction, teamwork, the provision of care, and clinical outcomes. In this way, wearables [28] can shift the burden of care from the NHS to the individual. Such a shift would represent greater convenience and independence for patients (as outlined earlier), while reducing costs and staff workloads. In fact, the NHS Long Term Plan has welcomed wearables from an efficiency standpoint [29], as the technology has the potential to revolutionize health care [28].

Remote patient monitoring, in the context of reducing the demand for health systems, has been of particular importance during the pandemic [30]. However, it should continue to retain its relevance [31] by reducing patient consultations [32] because of the health care sector’s focus on patient care and the versatility of wearables in catering to a wide spectrum of needs, from acting as a preventive tool in promoting fitness to managing chronic conditions [33].

#### Challenges Relating to Wearables

Although it has been stated that wearables can empower and emancipate patients [34] to manage their own care, the efficacy of these devices has attracted skepticism from some physicians [35], especially because the technology is emerging. However, change should be welcome, as patients are an “untapped resource” [7]. If patients were to take a more proactive role in their care, then the effects on the “quality and sustainability of health systems” could be transformative [7].

However, the accuracy of wearables is a concern that may deter their use, especially if they fail to produce reliable data. Therefore, regulatory oversight may be beneficial in ensuring that only accurate, tested devices are in circulation. Medical devices are regulated in the United Kingdom by the Medicines and Healthcare Products Regulatory Agency (MHRA) [36]. Nonetheless, certain wearables may not be regulated by the MHRA, as devices such as the Fitbit explicitly state that they are neither medical devices nor are “intended to diagnose, treat, cure or prevent any disease” [37]. Therefore, this may undermine the perceived efficacy of such devices and thereby fuel the skepticism of health care professionals. However, as wearables become more accurate, this is likely to change; some consumer-targeted wearables, such as Apple Watch, have already received Food and Drug Administration approval in the United States [38]. Consequently, it seems to be only a matter of time before approval is sought under the MHRA.

Furthermore, Accenture [39] advised that physicians should promote digital engagement and awareness of such devices among patients. This recommendation followed the findings that more than half of those surveyed [39] would take more responsibility for their care if their health care provider encouraged them to. However, only one-tenth of the respondents [39] reported having been recommended any digital tools to manage their care. It has been argued that despite initial reservations from patients, typically arising from a lack of confidence or knowledge, “it is incumbent on providers to foster [patients’] self-reliance” [7]. Clearly, with “self-management gaining ascendancy as a concept” [40,41], there is more to be done, including possibly reshaping the perceptions of providers and patients [7].

### Objectives

This study aims to identify, summarize, and synthesize knowledge to answer the following research question: “How can wearable health technology empower individuals to take greater responsibility for their health and care?” To the researcher’s knowledge, a review has yet to be conducted in this area; other reviews did not specifically focus their research on the concept of empowerment. Hence, research is needed to fill this gap and convey the importance of wearables to health care professionals.

## Methods

### Design

A scoping review design was chosen for its exploratory nature [42], which is useful when the international evidence base is heterogeneous [43]. In addition, this design enables the researcher to determine the range of available evidence and identify research gaps to guide future research [44].

Furthermore, the need to integrate research from a wide variety of sources and perspectives [43] across a broad area lends itself to a scoping review over alternative designs. A systematic review was found to be too restrictive and limited the materials considered [45,46], whereas research in the wearable field did not seem to place the same emphasis on theory as would be required for a realist review [47].

The 22-item PRISMA-ScR (Preferred Reporting Items for Systematic Reviews and Meta-Analyses extension for Scoping Reviews) [48] checklist was used, as it indicates what should be included in a scoping review. Background reading was conducted to ensure adherence to the latest guidelines. For example, there have been numerous additions [49-53] following the publication of a seminal paper by Arksey and O’Malley [44], which initially proposed a methodological framework for undertaking scoping reviews. The guidance document published by the Joanna Briggs Institute (JBI) [54] was also followed.

### Selection Criteria

Selection criteria were set to ensure the coverage of evidence, while excluding irrelevant papers. Hence, the inclusion criteria were as follows: English-language articles, a focus on wearables rather than other digital health technologies, and relevance to the research objective (by offering information that may relate to empowerment, such as barriers to use or discussions of the efficacy of certain wearables, even if such information had not been explicitly linked to empowerment). The researcher was selective in only including sources where there was a substantive focus on wearables rather than those that only mentioned wearables in passing. Regarding the inclusion of literature reviews, the individual studies of the review were screened. If many of these met the inclusion criteria, the review was included instead of the individual studies.

Despite wearable technology being a fast-moving area, no limits were imposed on the publication year of articles. Studies were excluded if the full text was unavailable or the studies were published outside of Europe or the United States. The latter was determined after preliminary searches indicated the presence of sufficient evidence. At this point, it was necessary to refine the selection criteria during the literature search phase because of practical constraints. In fact, Arksey and O’Malley [44] encouraged an iterative approach to research by using broad searches to first gain a *sense* of the field and thereafter setting any search parameters more strictly to meet the research requirements. Such an approach is further supported by the fact that “reading is central to reviewing literature” [55] and informing literature searches.

### Search Strategy

Database searches included MEDLINE, EMBASE, PsycINFO, HMIC, and Cochrane Library. Gray literature was also considered by searching OpenGrey, Google Scholar, and independent think tanks. The literature search was completed in early February 2021. A further search was conducted in May 2021 to account for any articles that may have been subsequently published.

The literature search involved relevant subject heading index terms, and subject headings were *exploded* as required. The search strategy (Multimedia Appendix 1) was adjusted to reflect variations in subject headings and syntax across the databases. For the breadth of coverage, a multipurpose search was used to search for keywords across numerous fields. A librarian was consulted to identify additional keywords.

Various strategies have been used to mitigate the risk of missing relevant evidence, including the use of synonymous terms, wildcard symbols, and truncation symbols. Boolean operators were used to combine the keywords and exclude others. Parentheses were used to group keywords joined by different Boolean operators, which yielded more relevant results than if a nesting approach had not been followed. In cases where quotation marks for phrase searching would potentially omit relevant results, proximity operators were used instead. The above-mentioned publication limits for language and location were also applied to the results. Furthermore, there was forward citation searching, and reference lists were snowballed for relevance to find studies that had not been identified in the initial literature search.

Duplicate records were identified using EndNote (Clarivate). The software-generated list of duplicates was manually reviewed to mitigate the risk of any records being incorrectly categorized as duplicates. The researcher then screened the remaining results and manually removed duplicates that had not been automatically flagged.

### Data Collection

The single researcher screened the literature by using a 2-step process, with a review of the title and abstract before the full text. If neither the title nor the abstract seemed relevant to the research, the article was excluded. If the title and abstract appeared relevant, the full-text article was read. Papers that did not meet the inclusion criteria were excluded, and the main reasons for exclusion were noted.

Scoping reviews do not typically address the appraisal of sources [44]. However, this would have resulted in a much larger sample size of evidence of questionable quality. Therefore, the JBI critical appraisal tool was used because of its relatively greater sensitivity to validity [56] to help ensure that any emergent findings would be based on high-quality evidence. This involved considering the limitations of the evidence, while assessing the congruity between the research aims, methodology, and findings [57].

### Data Charting

Key details were extracted to assess the relevance of a study [58], including publication details and study details relating to the objectives, findings, and type of wearable device. A data charting form (Multimedia Appendix 2) was adapted from the JBI [59] to incorporate other relevant details described elsewhere [60]. This form was piloted and updated with additional data that the researcher wished to chart.

### Data Analysis

Oftentimes, reviews fail to go beyond a summary of the evidence. Hence, this research followed the 6-step process of thematic analysis by Braun and Clarke [61], which involved familiarization with the data, coding of the data, generation of themes based on the codes, refinement of the themes, naming and defining the themes, and final write-up.

NVivo (QSR International) was used for a more structured analysis, as each source was individually uploaded and coded, which enabled the identification of themes from a wide evidence base. Themes were refined, with the findings being presented in the style of a narrative synthesis and related to the research question.

Such an approach to analysis and synthesis accords with guidance from Arksey and O’Malley [44], which stated the need for a scoping review to potentially use a “thematic construction in order to present a narrative account of existing literature.” This has been reflected in the PRISMA-ScR [48] and guidance on advancing the methodology of scoping reviews [49]. There are also examples of scoping reviews incorporating such an approach to analysis [62-64].

## Results

### Literature Search

**Figure 1:**
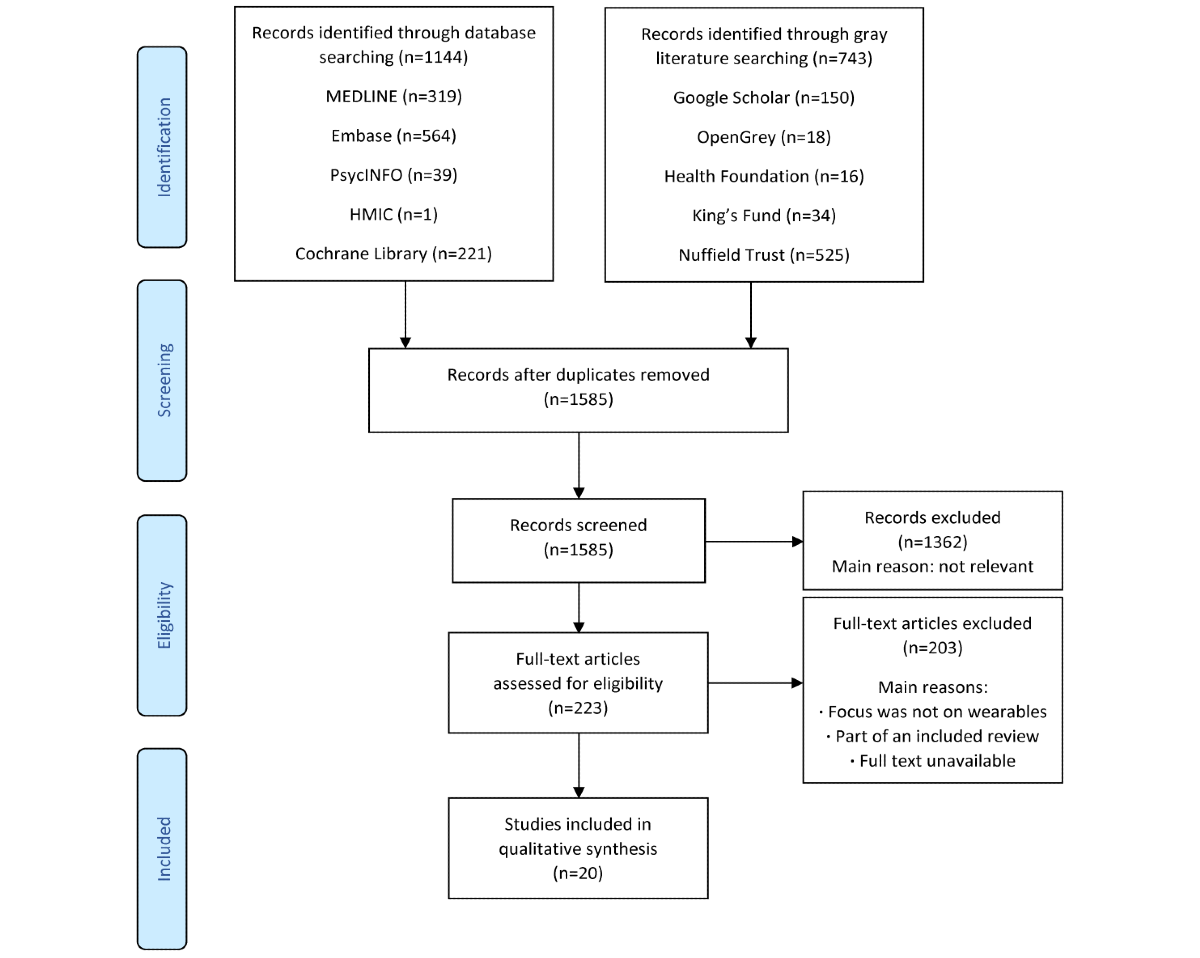
PRISMA (Preferred Reporting Items for Systematic Reviews and Meta-Analyses) flow diagram to illustrate the literature search.

The search (Figure 1) identified 1887 records. Following screening, 20 studies were included in the final data set, as summarized in Multimedia Appendix 3 [65-84]. Some of these studies were identified for inclusion in gray literature searches [77,78,83] or snowballing the reference lists of the included studies [76,80].

### Study Characteristics

The 20 included sources represent a significant body of literature, collectively accounting for >7000 participants. The studies were published between 2015 and 2021, with the number of studies appearing to have generally increased year-on-year. Most studies were published in the United States (12/20, 60%). The studies used quantitative (10/20, 50%), qualitative (8/20, 40%), and mixed methods (2/20, 10%; Multimedia Appendix 4). Funding details were provided by 65% (13/20) of the studies (Multimedia Appendix 5). Although Fitbit was the most common brand of wearable used (10/20, 50%), several studies (9/20, 45%) included multiple brands or discussed wearables in general.

## Discussion

A total of 3 main themes, relevant to user empowerment, emerged from the literature, namely, *Health Care Providers—Benefits and Involvement*, *Behavior Change*, and *Barriers to Use*. Multimedia Appendix 6 [65-84] lists the contributions of the included studies to each theme.

### Theme: Health Care Providers—Benefits and Involvement

#### Collaboration Between Providers and Patients

Health care providers are an important part of health care systems [85]. Therefore, it may be expected that providers would be considered as part of the literature on how wearables can empower patients.

Collaboration between research management and health care staff is imperative, especially during the study design process, as such a partnership may benefit patient compliance, particularly for those with cognitive impairments [65]. However, the role of clinicians may extend further. Outside of the research context, patients may rely on the clinicians’ acceptance of their decision to use a wearable device for other purposes, including as part of rehabilitation; hence, it would be incorrect to limit the role of health care professionals to simply prescribing medication without considering their role in educating patients [66]. The significance of such support and backing from clinicians may be easily overlooked.

Users seem to appreciate that consumer wearables are not medically accurate devices and that clinicians would not solely rely on data from such devices to make clinical decisions [84]. An open-minded, supportive approach may encourage patients to share data with their clinicians [84]. However, clinicians who are unwilling to engage with wearables and support their empowered patients, on the grounds of potential inaccuracies regarding data [84], may risk foregoing the benefits attributable to wearables.

#### Benefits to Providers and Patients

Wearables may offer several benefits to clinicians. First, wearables may offer objective, real-time patient data [66,76]. This would allow clinicians to remotely supervise progress [72,76] and provide comfort to patients who may otherwise feel that they are just communicating their subjective experiences and perceptions [76]. In such cases, it would be possible to use such data to inform clinicians of a patient’s history, thereby enabling a more personalized approach to treatment tailored to individual needs that can be adjusted according to the management plan [66,76]. This should enable more timely feedback so that clinicians can be more responsive to situational changes [80]. Access to data such as nutrition and activity-related information over an extended period may offer a solution to the issues of conventional health measurements and tests, as clinicians would benefit from a more complete picture of a patient’s health status [80]. In addition, data from wearables may eventually be used for risk stratification and early intervention [83], which should prevent further deterioration.

Furthermore, the accessibility of wearable data to patients may facilitate communication and assist with patient education [66]. Better-informed patients can offer more worthwhile contributions to any discussion, thereby promoting shared decision-making [66] and assisting with adherence to what is agreed [76,83]. In fact, a higher quality of life was associated with patients taking a more proactive role in their health [66]. There is the important caveat that to maximize these benefits, health care professionals should first identify patients with the willingness and ability to self-manage, especially because sustaining engagement can be challenging [83].

It is not difficult to imagine the potential for a large-scale rollout of wearables, which may help reduce the contact time and offer a more cost-effective approach to providers [75,76]. Such improvements in efficiency would likely free up resources, thereby alleviating the burden on health systems. The achievement of this is realistic, as supported by the Nuffield Trust [83], which has reported that “professional monitoring interventions for chronic conditions, whereby data is sent to the health care team, have had very positive results on health outcomes and resource use.”

Data from wearables can also be integrated into medical records to facilitate care [70,80], which can help overcome current barriers to reporting and retrieving data for inpatients and remote monitoring [80]. Patients living with chronic conditions often feel undersupported in managing their conditions [83]; therefore, wearables may offer this support. This is largely why wearables and other patient-facing technologies have been praised as a “bright hope” in the health care sector horizon [83].

#### Challenges to Wearables Advocation in the Health Care Sector

Certain health care services do not have the best track record for the uptake of technology. For example, in the United Kingdom, the NHS has been portrayed as “one of the most backward industries in responding to digital technology” [83]. The Nuffield Trust has captured the fact that the NHS has the potential to capitalize on consumer wearables [83]. However, consumer wearables may not be suitable for use, in their current state, by health care professionals. In fact, poorly calibrated devices can work counterproductively by worsening health outcomes and increasing staff workload [83]. Nevertheless, care should be taken not to be overly critical about the lack of accuracy of certain wearables because of benefits associated with aspects such as the provision of insights over extended periods [17].

Staff may require further upskilling to encourage engagement with wearables and facilitate behavior change [83]. This may demand professional monitoring and the provision of feedback on an ongoing basis [83]. In the short term, this may impose greater pressure on staff as it will add to workers’ responsibilities and may therefore appear unfeasible given the existing strain on staff. However, the short-term increase in workload may result in an overall reduction in workers’ commitments over the long term because of benefits associated with self-measurement of readings and the consequential reduction in appointments for such purposes [86].

Ultimately, providers have much to gain from patients taking steps to monitor their own health. To realize these benefits, health care professionals should encourage patients by adopting a supportive attitude, recognizing that wearables offer a means for patients to take a more proactive role in managing their health rather than viewing the devices too critically. In fact, diffusion of innovations theory [87] classifies adopters into categories, ranging from those who easily embrace change to *laggards* who are more skeptical about the innovation. Applying this theory [87] to the adoption of wearables, providers can play an important role in seeking to convince laggards about the benefits of wearables.

### Theme: Behavior Change

#### Overview

Breaking bad habits and establishing good ones, as part of a sustainable change to one’s lifestyle, requires positive actions whereby attitudes or behaviors may need to shift. The potential for wearables to draw on various behavior change techniques to prompt positive behavior change [20] holds promise for individuals willing to take greater responsibility for their health and care. Behavior change through wearables can take many forms, from reminders and positive reinforcement associated with progress tracking and reporting to social group support for motivational purposes. However, such aspects, among others, can also give rise to negative outcomes if not carefully catered for, as discussed in the following sections.

#### Behavior Change Techniques and Support

Continually providing information to users through wearables may be useful for consolidating patients’ understanding of their conditions and prompting behavior changes [66]. Furthermore, the ability of wearables to track progress and achievements could bolster adherence to exercise, which aligns with the behavior change theory [66]. It has also been suggested that introducing behavioral counseling based on feedback from wearables may lead to better results [70]. Another study has suggested the potential for activity trackers to complement behavioral counseling because of the behavior change techniques embedded in wearables, including those related to goal setting and social support [74]. These behavior change techniques have been leveraged by certain wearables [73,80] to help achieve positive changes, such as by promoting an active lifestyle [83]. Wearables seem to support behavior change, as another study has concluded that wearables further benefit patients in achieving their outcomes, as opposed to counseling alone [75].

Contrary to the position that has been taken in these studies, which have suggested that wearables can be effective, and the results for patients can be enhanced through the additional use of behavioral counseling, wearables’ value as a positive behavior change strategy may be context dependent. This is supported by a study that found that activity tracking was insufficient for improving pain-related outcomes or daily activity without behavior change support [72]. Despite not tracking changes in variables linked to behavior change theories, it has been argued that wearables may not be effective from a behavior change standpoint when promoting physical activity in college students [73].

In one study, only a few participants recognized specific behavior changes arising from the use of wearables [71]. These participants were more disciplined and conscious about activity levels and which exercises were more effective [71]. Although only a few commented on any behavior changes, the subjective nature of these changes may mean that others made similar progress but did not recognize such progress. Another study stated that their effect size for behavioral outcomes ranged between small and medium but could not identify which aspects of the devices resulted in this finding; instead, they speculated that this was because of greater intrinsic motivation for exercise [74].

An analysis of behavior change techniques used by activity trackers suggested that wearables commonly have more *controlling* features than those that promote autonomy [69]. For some users, this focus on rewards or social comparison may only appear detrimental to their physical activity in the long term [69] and may not be reflected in the findings of relatively short studies.

Moreover, physical activity levels seem to affect users’ perceptions of wearables, as those who are more active generally found the devices to have a higher number of *motivational affordances*, which refer to the features of technology that motivate and support users to meet their goals [79]. It has been suggested that this is because of greater familiarity with the motivational features of wearables, whereas novice exercisers may not understand or notice these features, such as the symbol denoting calorie burn [79]. Therefore, guided studies may not generalize to first-time, real-world use [79,80].

#### Self-efficacy

Self-efficacy refers to an individual’s belief that they can perform a task [88]. The strength of self-efficacy is important in influencing behavior change and how the individual responds to adversity [88].

Wearables appeared to draw on 3 sources of self-efficacy proposed by Bandura [88]; these have been credited with increasing user compliance and positive behavior change [77]. The first source relates to personal accomplishments [88], which are encompassed by the various features of wearable devices, including awards, progress toward activity goals, and performance over time [77,82]. The use of activity reminders forms part of the second source of self-efficacy, related to verbal persuasion [88], as motivational notifications can encourage users to progress and meet their goals [77,84]. The third source is termed “vicarious experience” [88] and links to the social aspects of wearables, whereby seeing users of a similar ability complete activities motivate certain users to believe that they can execute the same tasks [77].

However, it may be detrimental to self-efficacy when users believe that they are significantly underachieving relative to their peers [77]. Therefore, individuals should be matched to fellow users with whom they identify and who are successfully achieving their goals, as otherwise they may be discouraged [84]. Of course, this must be balanced with the privacy implications associated with personal data use, as individuals must be provided with transparent information about how their data will be used, coupled with data minimization techniques to ensure that only data required for the particular objective are being used and shared [89]. Nevertheless, designers should continue to consider sources of self-efficacy when developing features for wearables [77].

#### Contextual Factors

Importantly, users’ perceptions of self-efficacy seem context dependent [77]. The internal context comprises cognitive, behavioral, and emotional factors [77], whereas the external context considers factors outside the user’s control, such as the weather or time of the day [77]. The internal context is particularly important for self-efficacy, as it can either neutralize or compound a negative external context, meaning that users will either persevere in the face of adversity or stop using the wearable device [77]. In the interest of long-term behavior change and compliance, users should be supported to develop positive internal processes. For example, it would be valuable for wearables to be capable of adjusting their feedback based on the momentary state of the user [84] to reinforce their successes while supporting them through any difficulties in meeting targets.

Wearables offer a safe environment, as users can try to meet their goals even after repeatedly falling short; this establishes the intrinsic motivation to stay committed [84]. However, the support offered by wearables may need to be individualized to reflect the uniqueness of users’ personalities and priorities, which can factor into the affordances of wearables [80], as better engagement may convert to positive steps for behavior change. In addition, it is believed to foster self-efficacy, thereby supporting self-management [84]. For example, less conscientious individuals may require additional motivational support to assist with goal setting [79]. In addition, because self-set targets may not aid motivation, it may be beneficial for wearables to suggest feasible goals after monitoring the user [84]. Less agreeable users may respond better to increased support for their autonomy or greater transparency to build trust in the technology [79]. Introverts may prefer greater privacy, whereas extroverts may be more receptive to social aspects, such as comparing activities with others [79].

Users comparing their own data against expected standards may prompt positive behavior change [82], as not meeting such standards may lead to discomfort, referred to as cognitive dissonance [80]. The companion app plays an important role in enabling users to process information, as it visualizes and contextualizes their data [82]; this positively affects self-reported health metrics [82]. Of course, the privacy implications, as discussed earlier, of identifying peer comparators with respect to expected standards must still be observed.

#### Incentivization

Economic incentives, such as offering discounts on insurance premiums or wellness products, also appear to increase the willingness of individuals to use wearables [81]. Such an approach, in terms of offering discounts, has been undertaken by health and life insurance providers who are motivated to minimize claims on their issued policies. For example, Vitality offers a discount on Apple Watch [90] and encourages members to track their activities via the app.

Consequently, incentivizing uptake may facilitate behavior change through regular use, but this would seem to be contingent on users’ satisfaction with the data privacy and technical provisions of the wearable device. Therefore, it is important to address any barriers so that they do not hinder the use of wearables and prevent users from beginning the process of positive behavior change.

#### Motivational Profile

The subsequent discussion on barriers to use centers primarily on the design of the wearable, among other factors. However, there may be a case for considering the motivational profile (degree of autonomy and motivation) of users [69] and the motivational affordances of devices [79] when using wearables as a tool for empowerment, as is evident that there may be contextual factors that affect the ability of wearables to inspire behavior change.

For wearables to empower individuals, it would be worth undertaking a preliminary assessment of individuals who may require additional support in the form of behavioral counseling. This will help ensure that patients receive appropriate support, as individuals whose motivational profiles are not matched to the wearable device may become demotivated and experience negative emotions from persistently failing to meet goals [69].

### Theme: Barriers to Use

Barriers to the adoption and use of wearables could have significant ramifications for empowerment.

Although individuals expressed willingness to use wearables, use seemed to be inconsistent; a study reported that >90% of the participants suspended use [65]. As this is not an isolated case, with the issue of compliance mentioned elsewhere [68], it is worth considering factors that may have contributed to this.

The barriers to use that were identified [65] include forgetting to apply, hospitalization, loss of interest, and temporary loss of the wearable device. Aside from the concerns of wearability, accuracy, and price, feelings of fatigue stemming from the use of technology highlight the need for wearables to constantly engage users, as loss of interest is a key reason for disuse [73]. It is perhaps surprising that losing wearables does not seem to be uncommon; this is evidenced by other studies [68,72,73], some of which have also reported malfunctioning devices that require replacement by the manufacturer [68].

#### Design-Related Aspects

In addition, although certain design aspects, such as color and size, may inﬂuence use [65], an aesthetically pleasing appearance may be a more important consideration for younger individuals [76].

Concerns regarding stigma arising from the use of certain wearables have also been raised. For instance, children who are overweight that wear the *badge* of an activity tracker may be bullied [91]. Similarly, this seems to factor into the decisions of patients who would prefer a sleek, discreet device rather than one that is overtly medical [76].

#### Technical Aspects

The technology itself may deter use. A study [67] has added the following to the list of potential barriers: health difficulties, technical difficulties, a lack of personalized advice, and an inability to track other types of physical activity such as strength exercises. These clearly represent barriers, as reported elsewhere [76]. Such concerns may also discourage regular use over a prolonged period [76], especially if individuals come to perceive that these issues are associated with all wearables.

Annoyances may also prevent users from engaging with the technology [71]. For example, users may be frustrated by the perceived inaccuracies of sleep or pulse monitors [71], as some have stopped using wearables for being unreliable [84]. Device inaccuracies have been cited elsewhere together with issues related to battery life [72].

#### Barriers That Are More Common for Older Users

In addition, a lack of familiarity [71] or not being tech-savvy [84] may mean that some individuals are put off by wearables that appear too complicated at first use. Such difficulties may be more common among the older generation [79], in the context of connecting wearables to smartphones and accessing metrics [71]. In fact, not owning a smartphone, through which many wearables tend to display such metrics, seemed to limit interest in tracking activities altogether [71].

Certain other barriers seem to apply to an older user base. Devices that require a high level of manual dexterity to operate proved unsuitable for older individuals to easily use [72,79]. Another complaint was that the displayed text was too small to read easily [79]. Furthermore, many users were frustrated by the lack of availability of instructions and guides for the execution of basic tasks. This may be more of an issue in research studies, as users typically have access to any device manual when they make a purchase themselves. However, technical issues are common and tend to be resolved by the staff leading the research study [72].

#### Cost

Cost may be another barrier, as even relatively low-cost trackers may be inaccessible to older adults [72]. For others, the cost is a nonissue, as it was suggested that if the device is beneficial, then it is a matter of answering the question, “What’s my health worth to me?” [76]. This highlights the possible need for individuals to weigh the advantages offered by a wearable device against its shortcomings to ascertain whether the device is of value and justifies the investment in one’s health.

Importantly, wearables should not seek to widen the health inequalities that have worsened during the COVID-19 pandemic [92], especially for the poorest in society who tend to be in the greatest need of care but least likely to receive such care [93]. Therefore, wearables should serve as an additional option for individuals to proactively manage their health care rather than acting as a replacement for any traditional mode of delivery.

#### Barriers Arising From Long-term Use

The nature of wearables, as a newly emerging technology that has gained traction in recent years, warrants further research and development [78] to allay concerns surrounding durability, comfort, power consumption, standardization, interoperability, accuracy, privacy, and conﬁdentiality. These potential issues are more likely to arise from regular, long-term use of wearables; however, they are often missed in shorter clinical studies [78]. If the barriers and concerns that have been raised are deemed by users to outweigh the benefits offered by the wearable, then this may discourage individuals from using such devices to monitor their health, thereby potentially interfering with their ability to follow an active lifestyle [80].

#### Privacy

Moreover, privacy concerns have often been raised [84]. This is illustrated by the recent acquisition of Fitbit by Google [94], which gave rise to concerns about how personal and health data were going to be used by a *tech giant* that is active in the AdTech and data commercialization fields [95]. Consequently, it is necessary to balance privacy and security concerns with potential benefits to users and the health system [80].

Another high-profile example of significant privacy concerns from the use of portable technology in the context of health care has arisen from the development and use of COVID Track and Trace apps around the world [96-98]. Although this does not fall within the strict definition of a wearable, the privacy concerns raised [99] with respect to the apps with regard to location tracking of individuals and the sharing and aggregation of personal data are equally applicable to the use of wearables that capture and process such types of user data.

#### Technology-Specific or General Barriers

It must be acknowledged that some of the criticisms of wearables that seem to hinder use could be specific to the brand of wearables used in a study. Therefore, although the aforementioned concerns should be considered, it is important to distinguish the specific nature of some barriers rather than applying them to wearables in general. For example, the inability to measure strength exercises appears to be specific to the wearable used in a study as part of a review [67]. In reality, the availability of a range of wearables, some of which are designed to track strength exercises, may present less of a barrier to use.

However, the fact that the aforementioned barriers have been described in the literature seems to suggest that such issues are prevalent rather than being restricted to a single brand of wearable technology, as Multimedia Appendix 3 shows the diversity of wearables included in this review. In addition, the barriers are significant and clearly need to be overcome to avert any further negative effects on user perceptions, which may otherwise discourage the use of wearables. Failure to take appropriate steps for damage control may erode public trust in wearables, thereby limiting the potential to empower new users to manage their health more proactively. Therefore, although all technologies seem to have their own shortcomings or barriers, issues relating to wearable health technology may be viewed more critically, as such wearables can inform decisions related to one’s health and care.

### Principal Findings

A summary of the principal findings with respect to these themes is provided in the following sections.

#### Health Care Providers—Benefits and Involvement

Providers play an important role in empowering patients to use wearables. Therefore, providers require support because of the short-term resource constraints that they are likely to face. However, data from wearables may help create a more holistic understanding of a patient’s health status, thereby accelerating the delivery of personalized advice. Better-informed patients should aid in communication and improve their adherence to advice.

#### Behavior Change

Wearables may lead to positive behavior changes. This may arise from the ability to set goals, receive motivational reminders, track progress, and contextualize user data via a companion app to facilitate understanding. Furthermore, peer comparison of activity data may benefit some in meeting their goals but may be detrimental to those who become discouraged from feeling that they are underperforming relative to their peers. Ultimately, wearables may better empower individuals by offering tailored support with positive reinforcement of users’ successes while encouraging users when they fail to meet their targets.

#### Barriers to Use

Barriers to user empowerment include a perceived lack of accuracy and overly complex devices. However, lack of accessibility may be a greater issue, with concerns about pricing and how not owning a smartphone may mean that individuals miss out on the interpretation of data facilitated by the companion app. Another major concern relates to privacy, in which wearables collect sensitive health data. Consequently, strategies are required to mitigate the associated risks.

### Strengths and Limitations

This review has its limitations. The nature of this research and its focus on wearable technology as a broad area may mean that relevant studies have been inadvertently missed. For example, although gray literature may reduce publication bias, it may give rise to selection bias because there is no gold standard method for retrieval [100]. Another potential source of bias may be the use of judgment when selecting studies for inclusion. Furthermore, the selection criteria may have excluded populations from low- and middle-income countries, where wearables can also be of benefit.

In certain circumstances, literature reviews have been included without including individual studies for review. It is important to note the reliance on the analysis undertaken as part of these reviews and that those reviews should be read alongside this review to see the full picture. Although this approach has its shortcomings from an analytic perspective, it was more practical as the individual studies that were screened met the inclusion criteria. It is also worth noting that although some studies that formed part of the literature review were identified from the initial literature searches for this scoping review, others were not. Although this only became apparent when the reference lists of the literature reviews were cross-checked against the records collated in the EndNote library, it gives rise to the question of how many other potential studies may have been missed and why?

The researcher took steps to minimize any bias and its effect on the research findings. The researcher consulted with senior academics throughout the research. A librarian guided the search strategy. Moreover, the researcher adhered to best practice recommendations from the PRISMA-ScR checklist and appraised the literature (which is not a requirement of scoping reviews) to further strengthen the rigor of this research. In fact, the very act of acknowledging these limitations has enabled the reader to contextualize the findings of the research within its limitations while demonstrating compliance with the recommended practice documented by the PRISMA-ScR [50].

The main consideration for this review was to balance the practicalities of research as a single researcher with the need to review representative, relevant evidence. This is where feedback on the research protocol and the availability of published scoping reviews (particularly those cited in the PRISMA-ScR “Tip Sheets” [48] as examples to illustrate good practice) have helped develop the methodology. Consequently, this review has been successful in meeting its aims and answering the research question; therefore, it should serve as a meaningful contribution to the literature in a dynamic, emerging area.

### Conclusions

Although this scoping review has its limitations, its value is underscored by the fact that it fills a gap in the literature by addressing the research question and aims.

Considerable literature findings support the proposition that wearable health technology can empower users and, in turn, benefit providers and patients. Even if patients are unable to entirely self-manage their conditions, wearables have the potential to empower users to take more responsibility for their health and inspire positive behavior changes.

However, the ability of wearables to empower users may be limited by several factors. To maximize the potential for consumer wearables to integrate with the health system, support from health care professionals is critical. In addition, user feedback should be considered with respect to common barriers to use, such as technical issues and privacy concerns. As part of this process, designers of wearables should seek to incorporate more personalized support by way of positive reinforcement of any successes alongside encouragement for users who fail to meet their targets.

Future research may report whether there has been any progress in overcoming the barriers to use, including those mentioned earlier and others raised as part of this review. Further investigation of the long-term effects of wearables on individuals’ outcomes through larger studies is warranted, as much of the literature revolves around small-scale studies. Moreover, despite the abundance of literature on wearables, what seems to be missing is the focus on the people who wear them. This may be because wearables, as viable instruments to assist with health care, have only been introduced in recent years. Specifically, future research may focus more closely on wearables and empowerment, especially as technology continues to evolve and advance over time. However, the challenge is for the publication of research to keep pace with rapid developments related to wearable health technology.

The adoption of wearables in the health sector may be gradual and fraught with challenges [101], but strategic change is certainly possible. In particular, any communication to relevant parties should emphasize the fact that although it may not be immediately apparent, each party has much to gain in the long run. Patients and users are expected to exercise greater control over their health and care decisions. Designers of the devices should benefit from having a more engaged user base. Similarly, individuals taking a more proactive role in their care should lessen the burden on clinicians and ease the pressure on the wider health system.

## Appendix

Multimedia Appendix 1 Search strategies for databases and gray literature.

Multimedia Appendix 2 Data charting form.

Multimedia Appendix 3 Summary of the included studies.

Multimedia Appendix 4 Characteristics of the included studies.

Multimedia Appendix 5 Funding details of the relevant included studies.

Multimedia Appendix 6 Contribution of the included studies to each of the themes.

## Abbreviations

ECG: electrocardiogram
JBI: Joanna Briggs Institute
MHRA: Medicines and Healthcare Products Regulatory Agency
NHS: National Health Service
PHI: participatory health informatics
PRISMA-ScR: Preferred Reporting Items for Systematic Reviews and Meta-Analyses extension for Scoping Reviews
