# Supplementary material for: Wearing the Future—Wearables to Empower Users to Take Greater Responsibility for Their Health and Care: Scoping Review
Source: JMIR Mhealth Uhealth. 2022 Jul 13;10(7):e35684. doi: 10.2196/35684 (PMC9330198; doi:10.2196/35684)
Supplement: Multimedia Appendix 1 [file mhealth_v10i7e35684_app1.pdf]

MEDLINE

Database: Ovid MEDLINE(R) and Epub Ahead of Print, In-Process & Other Non-Indexed Citations and Daily <1946 to January 26, 2021>

Search Strategy:

1 wearable electronic devices/ or fitness trackers/ or smart glasses/ (3889)

2 ((wearable or body mounted) adj2 (device$ or sensor$ or monitor$ or tech$ or biosensor$ or gadget$)).mp. [mp=title, abstract, original title, name of substance word, subject heading word, floating sub-heading word, keyword heading word, organism supplementary concept word, protocol supplementary concept word, rare disease supplementary concept word, unique identifier, synonyms] (10151)

3 ((smart or google) adj2 (watch$ or jewellery or wrist band$ or wristband$ or tracker$ or accessory or accessories)).mp. (175)

4 smart ring$1.mp. (5)

5 smartwatch.mp. (310)

6 (fitness tracker$ or activity tracker$ or wearable ECG).mp. [mp=title, abstract, original title, name of substance word, subject heading word, floating sub-heading word, keyword heading word, organism supplementary concept word, protocol supplementary concept word, rare disease supplementary concept word, unique identifier, synonyms] (1393)

7 ((Apple or microsoft) adj2 watch$).mp. [mp=title, abstract, original title, name of substance word, subject heading word, floating sub-heading word, keyword heading word, organism

supplementary concept word, protocol supplementary concept word, rare disease supplementary concept word, unique identifier, synonyms] (149)

8 (fitbit$ or "Omron heart guide" or "samsung gear 2" or "get active slim" or "jawbone UP" or "Google Glass" or GoQii or HoloLens or garmin).mp. (1156)

9 (biometric adj3 (watch$ or jewellery or wearable or wrist band$ or wristband$ or tracker$)).mp. [mp=title, abstract, original title, name of substance word, subject heading word, floating sub-heading word, keyword heading word, organism supplementary concept word, protocol supplementary concept word, rare disease supplementary concept word, unique identifier, synonyms] (16)

10 or/1-9 (12157)

11 exp attitude to health/ or health knowledge, attitudes, practice/ (427812)

12 consumer behavior/ or sedentary behavior/ (32211)

13 (self manage$ or self care or responsib$ or own care or empower$).mp. [mp=title, abstract, original title, name of substance word, subject heading word, floating sub-heading word, keyword heading word, organism supplementary concept word, protocol supplementary concept word, rare disease supplementary concept word, unique identifier, synonyms] (638316)

14 (health adj2 (attitude$ or knowledge$ or practice or behavio$)).mp. [mp=title, abstract, original title, name of substance word, subject heading word, floating sub-heading word, keyword heading word, organism supplementary concept word, protocol supplementary concept word, rare disease supplementary concept word, unique identifier, synonyms] (383401)

15 ((consumer$ or patient$) adj2 (attitude$ or knowledge$ or practice or behavio$)).mp. [mp=title, abstract, original title, name of substance word, subject heading word, floating sub-heading word, keyword heading word, organism supplementary concept word, protocol supplementary concept word, rare disease supplementary concept word, unique identifier, synonyms] (59492)

16 (health adj2 (attitude$ or knowledge or practice$ or behavio$)).mp. (387462)

17 sedentary behavio$.mp. (13938)

18 (lifestyle$ or life style$).mp. [mp=title, abstract, original title, name of substance word, subject heading word, floating sub-heading word, keyword heading word, organism supplementary concept word, protocol supplementary concept word, rare disease supplementary concept word, unique identifier, synonyms] (146093)

19 (personali#ation or medtech or healthtech or med tech or health tech).mp. (3114)

20 ((personalised or personalized) adj2 (health or medic$ or wellness or wellbeing)).mp. (16992)

21 or/11-20 (1392229)

22 10 and 21 (1612)

23 exp Europe/ (1443398)

24 (Europe$ or andorra or Austria$ or belgium or Albania$ or Estonia$ or Latvia$ or Lithuania$ or "bosnia and herzegovina" or Bosnian or Bulgaria$ or Croatia$ or czech republic or hungary or Hungarian or kosovo or "republic of north macedonia" or moldova or montenegro or poland or polish or "republic of belarus" or Romania$ or Serbia$ or Slovakia$ or Slovenia$ or

ukraine or france or French or german$ or gibraltar or united kingdom or channel islands or england or English or ireland or irish or scotland or Scottish or wales or welch or Britain or british or greece or italy or Italian or liechtenstein or luxembourg or cyprus or malta or sicily or monaco or netherlands or dutch or portugal or portuguese or san marino or denmark or Danish or Iceland$ or norway or Norwegian or sweden or Swedish or spain or spanish or switzerland or swiss or Armenia$ or azerbaijan or Georgia$ or ussr or Russia$ or kazakhstan or kyrgyzstan or Uzbekistan or balkan).mp. (3652056)

25 exp United States/ (1369297)

26 (United States or USA or North America$).mp. (1213354)

27 (united states or alabama or georgia or kentucky or maryland or new york or north carolina or ohio or pennsylvania or south carolina or tennessee or virginia or west virginia or illinois or indiana or michigan or minnesota or wisconsin or delaware or "district of columbia" or iowa or kansas or missouri or nebraska or north dakota or oklahoma or south dakota or connecticut or maine or massachusetts or new hampshire or rhode island or vermont or idaho or montana or oregon or washington or wyoming or alaska or california or hawaii or arkansas or florida or louisiana or mississippi or arizona or colorado or nevada or new mexico or texas or utah).mp. (1655550)

28 or/23-27 (5309248)

29 22 and 28 (319)

Embase

Database: Embase <1974 to 2021 January 26>

Search Strategy:

1 exp wearable computer/ or exp smart watch/ (4482)

2 exp activity tracker/ (3445)

3 smart glasses/ (86)

4 ((wearable or body mounted) adj2 (device$ or sensor$ or monitor$ or tech$ or biosensor$ or gadget$)).mp. [mp=title, abstract, heading word, drug trade name, original title, device manufacturer, drug manufacturer, device trade name, keyword, floating subheading word, candidate term word] (9797)

5 ((smart or google) adj2 (watch$ or jewellery or wrist band$ or wristband$ or tracker$ or accessory or accessories)).mp. (365)

6 smart ring$1.mp. (4)

7 smartwatch.mp. (387)

8 (fitness tracker$ or activity tracker$ or wearable ECG).mp. [mp=title, abstract, heading word, drug trade name, original title, device manufacturer, drug manufacturer, device trade name, keyword, floating subheading word, candidate term word] (1593)

9 ((Apple or microsoft) adj2 watch$).mp. [mp=title, abstract, heading word, drug trade name, original title, device manufacturer, drug manufacturer, device trade name, keyword, floating subheading word, candidate term word] (221)

10 (fitbit$ or "Omron heart guide" or "samsung gear 2" or "get active slim" or "jawbone UP" or "Google Glass" or GoQii or HoloLens or garmin).mp. (1880)

11 (biometric adj3 (watch$ or jewellery or wearable or wrist band$ or wristband$ or tracker$)).mp. [mp=title, abstract, heading word, drug trade name, original title, device manufacturer, drug manufacturer, device trade name, keyword, floating subheading word, candidate term word] (19)

12 or/1-11 (15472)

13 attitude to health/ (115685)

14 consumer attitude/ (5279)

15 sedentary lifestyle/ (15159)

16 (self manage$ or self care or responsib$ or own care or empower$).mp. [mp=title, abstract, heading word, drug trade name, original title, device manufacturer, drug manufacturer, device trade name, keyword, floating subheading word, candidate term word] (800326)

17 (health adj2 (attitude$ or knowledge$ or practice or behavio$)).mp. [mp=title, abstract, heading word, drug trade name, original title, device manufacturer, drug manufacturer, device trade name, keyword, floating subheading word, candidate term word] (300632)

18 ((consumer$ or patient$) adj2 (attitude$ or knowledge$ or practice or behavio$)).mp. [mp=title, abstract, heading word, drug trade name, original title, device manufacturer, drug manufacturer, device trade name, keyword, floating subheading word, candidate term word] (127030)

19 (health adj2 (attitude$ or knowledge or practice$ or behavio$)).mp. (305380)

20 sedentary behavio$.mp. (9048)

21 (lifestyle$ or life style$).mp. [mp=title, abstract, heading word, drug trade name, original title, device manufacturer, drug manufacturer, device trade name, keyword, floating subheading word, candidate term word] (232524)

22 (personali#ation or medtech or healthtech or med tech or health tech).mp. (4786)

23 ((personalised or personalized) adj2 (health or medic$ or wellness or wellbeing)).mp. (58861)

24 or/13-23 (1442919)

25 12 and 24 (2359)

26 exp Europe/ (1607256)

27 (Europe$ or andorra or Austria$ or belgium or Albania$ or Estonia$ or Latvia$ or Lithuania$ or "bosnia and herzegovina" or Bosnian or Bulgaria$ or Croatia$ or czech republic or hungary or Hungarian or kosovo or "republic of north macedonia" or moldova or montenegro or poland or polish or "republic of belarus" or Romania$ or Serbia$ or Slovakia$ or Slovenia$ or ukraine or france or French or german$ or gibraltar or united kingdom or channel islands or england or English or ireland or irish or scotland or Scottish or wales or welch or Britain or british or greece or italy or Italian or liechtenstein or luxembourg or cyprus or malta or sicily or monaco or netherlands or dutch or portugal or portuguese or san marino or denmark or Danish or Iceland$ or norway or Norwegian or sweden or Swedish or spain or spanish or switzerland or swiss or Armenia$ or azerbaijan or Georgia$ or ussr or Russia$ or kazakhstan or kyrgyzstan or Uzbekistan or balkan).mp. (3551023)

28 exp United States/ (1246253)

29 (United States or USA or North America$).mp. (2226941)

30 united states/ or alabama/ or georgia/ or kentucky/ or maryland/ or new york/ or north carolina/ or ohio/ or pennsylvania/ or south carolina/ or tennessee/ or virginia/ or west virginia/ or illinois/ or indiana/ or michigan/ or minnesota/ or wisconsin/ or delaware/ or "district of columbia"/ or iowa/ or kansas/ or missouri/ or nebraska/ or north dakota/ or oklahoma/ or south dakota/ or connecticut/ or maine/ or massachusetts/ or new hampshire/ or rhode island/ or vermont/ or idaho/ or montana/ or oregon/ or washington/ or wyoming/ or alaska/ or california/ or hawaii/ or arkansas/ or florida/ or louisiana/ or mississippi/ or arizona/ or colorado/ or nevada/ or new mexico/ or texas/ or utah/ (1245484)

31 (united states or alabama or georgia or kentucky or maryland or new york or north carolina or ohio or pennsylvania or south carolina or tennessee or virginia or west virginia or illinois or indiana or michigan or minnesota or wisconsin or delaware or "district of columbia" or iowa or kansas or missouri or nebraska or north dakota or oklahoma or south dakota or connecticut or maine or massachusetts or new hampshire or rhode island or vermont or idaho or montana or oregon or washington or wyoming or alaska or california or hawaii or arkansas or florida or louisiana or mississippi or arizona or colorado or nevada or new mexico or texas or utah).mp. (2360451)

32 or/26-31 (5764492)

33 25 and 32 (564)

PsycINFO

Database: APA PsycInfo <1967 to January Week 3 2021>

Search Strategy:

1 ((wearable or body mounted) adj2 (device$ or sensor$ or monitor$ or tech$ or biosensor$ or gadget$)).mp. [mp=title, abstract, heading word, table of contents, key concepts, original title, tests & measures, mesh] (915)

2 ((smart or google) adj2 (watch$ or jewellery or wrist band$ or wristband$ or tracker$ or accessory or accessories)).mp. (32)

3 smart ring$1.mp. (1)

4 smartwatch.mp. (72)

5 (fitness tracker$ or activity tracker$ or wearable ECG).mp. [mp=title, abstract, heading word, table of contents, key concepts, original title, tests & measures, mesh] (171)

6 ((Apple or microsoft) adj2 watch$).mp. [mp=title, abstract, heading word, table of contents, key concepts, original title, tests & measures, mesh] (18)

7 (fitbit$ or "Omron heart guide" or "samsung gear 2" or "get active slim" or "jawbone UP" or "Google Glass" or GoQii or HoloLens or garmin).mp. (251)

8 (biometric adj3 (watch$ or jewellery or wearable or wrist band$ or wristband$ or tracker$)).mp. [mp=title, abstract, heading word, table of contents, key concepts, original title, tests & measures, mesh] (1)

9 or/1-8 (1261)

10 health attitudes/ or health knowledge/ (17260)

11 exp health behavior/ (35858)

12 consumer behavior/ (29622)

13 sedentary behavior/ (1721)

14 (self manage$ or self care or responsib$ or own care or empower$).mp. [mp=title, abstract, heading word, table of contents, key concepts, original title, tests & measures, mesh] (168606)

15 (health adj2 (attitude$ or knowledge$ or practice or behavio$)).mp. [mp=title, abstract, heading word, table of contents, key concepts, original title, tests & measures, mesh] (94879)

16 ((consumer$ or patient$) adj2 (attitude$ or knowledge$ or practice or behavio$)).mp. [mp=title, abstract, heading word, table of contents, key concepts, original title, tests & measures, mesh] (68478)

17 (health adj2 (attitude$ or knowledge or practice$ or behavio$)).mp. (96830)

18 sedentary behavio$.mp. (3040)

19 (lifestyle$ or life style$).mp. [mp=title, abstract, heading word, table of contents, key concepts, original title, tests & measures, mesh] (39753)

20 (personali#ation or medtech or healthtech or med tech or health tech).mp. (2730)

21 ((personalised or personalized) adj2 (health or medic$ or wellness or wellbeing)).mp. (1190)

22 or/10-19 (351123)

23 9 and 22 (260)

24 (Europe$ or andorra or Austria$ or belgium or Albania$ or Estonia$ or Latvia$ or Lithuania$ or "bosnia and herzegovina" or Bosnian or Bulgaria$ or Croatia$ or czech republic or hungary or Hungarian or kosovo or "republic of north macedonia" or moldova or montenegro or poland or polish or "republic of belarus" or Romania$ or Serbia$ or Slovakia$ or Slovenia$ or ukraine or france or French or german$ or gibraltar or united kingdom or channel islands or england or English or ireland or irish or scotland or Scottish or wales or welch or Britain or british or greece or italy or Italian or liechtenstein or luxembourg or cyprus or malta or sicily or monaco or netherlands or dutch or portugal or portuguese or san marino or denmark or Danish or Iceland$ or norway or Norwegian or sweden or Swedish or spain or spanish or switzerland or swiss or Armenia$ or azerbaijan or Georgia$ or ussr or Russia$ or kazakhstan or kyrgyzstan or Uzbekistan or balkan).mp. (543708)

25 (United States or USA or North America$).mp. (195976)

26 (united states or alabama or georgia or kentucky or maryland or new york or north carolina or ohio or pennsylvania or south carolina or tennessee or virginia or west virginia or illinois or indiana or michigan or minnesota or wisconsin or delaware or "district of columbia" or iowa or kansas or missouri or nebraska or north dakota or oklahoma or south dakota or connecticut or maine or massachusetts or new hampshire or rhode island or vermont or idaho or montana or oregon or washington or wyoming or alaska or california or hawaii or arkansas or florida or louisiana or mississippi or arizona or colorado or nevada or new mexico or texas or utah).mp. (368210)

27 or/24-26 (881110)

28 23 and 27 (39)

Healthcare Management Information Consortium

Database: HMIC Health Management Information Consortium <1979 to November 2020>

Search Strategy:

1 ((wearable or body mounted) adj2 (device$ or sensor$ or monitor$ or tech$ or biosensor$ or gadget$)).mp. [mp=title, other title, abstract, heading words] (10)

2 ((smart or google) adj2 (watch$ or jewellery or wrist band$ or wristband$ or tracker$ or accessory or accessories)).mp. (0)

3 smart ring$1.mp. (0)

4 smartwatch.mp. (1)

5 (fitness tracker$ or activity tracker$ or wearable ECG).mp. [mp=title, other title, abstract, heading words] (5)

6 ((Apple or microsoft) adj2 watch$).mp. [mp=title, other title, abstract, heading words] (0)

7 (fitbit$ or "Omron heart guide" or "samsung gear 2" or "get active slim" or "jawbone UP" or "Google Glass" or GoQii or HoloLens or garmin).mp. (4)

8 (biometric adj3 (watch$ or jewellery or wearable or wrist band$ or wristband$ or tracker$)).mp. [mp=title, other title, abstract, heading words] (0)

9 or/1-8 (18)

10 health behaviour/ or life style/ (2531)

11 sedentary life/ (169)

12 active life/ (59)

13 (self manage$ or self care or responsib$ or own care or empower$).mp. [mp=title, other title, abstract, heading words] (17885)

14 (health adj2 (attitude$ or knowledge$ or practice or behavio$)).mp. [mp=title, other title, abstract, heading words] (4853)

15 ((consumer$ or patient$) adj2 (attitude$ or knowledge$ or practice or behavio$)).mp. [mp=title, other title, abstract, heading words] (2530)

16 (health adj2 (attitude$ or knowledge or practice$ or behavio$)).mp. (5119)

17 sedentary behavio$.mp. (143)

18 (lifestyle$ or life style$).mp. [mp=title, other title, abstract, heading words] (4059)

19 (personali#ation or medtech or healthtech or med tech or health tech).mp. (746)

20 ((personalised or personalized) adj2 (health or medic$ or wellness or wellbeing)).mp. (78)

21 or/10-20 (28678)

22 9 and 21 (7)

23 exp Europe/ (59490)

24 (Europe$ or andorra or Austria$ or belgium or Albania$ or Estonia$ or Latvia$ or Lithuania$ or "bosnia and herzegovina" or Bosnian or Bulgaria$ or Croatia$ or czech republic or hungary or Hungarian or kosovo or "republic of north macedonia" or moldova or montenegro or poland or polish or "republic of belarus" or Romania$ or Serbia$ or Slovakia$ or Slovenia$ or ukraine or france or French or german$ or gibraltar or united kingdom or channel islands or england or English or ireland or irish or scotland or Scottish or wales or welch or Britain or british or greece or italy or Italian or liechtenstein or luxembourg or cyprus or malta or sicily

or monaco or netherlands or dutch or portugal or portuguese or san marino or denmark or Danish or Iceland$ or norway or Norwegian or sweden or Swedish or spain or spanish or switzerland or swiss or Armenia$ or azerbaijan or Georgia$ or ussr or Russia$ or kazakhstan or kyrgyzstan or Uzbekistan or balkan).mp. (82906)

25 exp United States of America/ (16563)

26 (United States or USA or North America$).mp. (19845)

27 (united states or alabama or georgia or kentucky or maryland or new york or north carolina or ohio or pennsylvania or south carolina or tennessee or virginia or west virginia or illinois or indiana or michigan or minnesota or wisconsin or delaware or "district of columbia" or iowa or kansas or missouri or nebraska or north dakota or oklahoma or south dakota or connecticut or maine or massachusetts or new hampshire or rhode island or vermont or idaho or montana or oregon or washington or wyoming or alaska or california or hawaii or arkansas or florida or louisiana or mississippi or arizona or colorado or nevada or new mexico or texas or utah).mp. (20615)

28 or/23-27 (118992)

29 22 and 28 (1)

Cochrane Library

ID Search Hits

#1 MeSH descriptor: [Wearable Electronic Devices] this term only 82

#2 MeSH descriptor: [Fitness Trackers] this term only 101

#3 MeSH descriptor: [Smart Glasses] this term only 2

#4 (wearable or body mounted) NEAR/2 (device* or sensor* or monitor* or tech* or biosensor* or gadget*) 3219

#5 (smart or google) NEAR/2 (watch* or jewellery or wrist NEXT band* or wristband* or tracker* or accessory or accessories) 51

#6 "smart ring" or "smart rings" 0

#7 smartwatch 53

#8 (fitness NEXT tracker* or activity NEXT tracker* or "wearable ECG") 498

#9 (Apple or microsoft) NEAR/2 watch* 41

#10 fitbit* or "Omron heart guide" or "samsung gear 2" or "get active slim" 508

#11 "jawbone UP" or "Google Glass" or GoQii or HoloLens or garmin 102

#12 biometric NEAR/3 (watch* or jewellery or wearable or wrist NEXT band* or wristband* or tracker*) 1

#13 #1 or #2 or #3 or #4 or #5 or #6 or #7 or #8 or #9 or #10 or #11 or #12 4133

#14 MeSH descriptor: [Attitude to Health] explode all trees 35652

#15 MeSH descriptor: [Health Knowledge, Attitudes, Practice] this term only 5993

#16 MeSH descriptor: [Consumer Behavior] this term only 821

#17 MeSH descriptor: [Sedentary Behavior] this term only 1129

#18 self NEXT manage* or "self care" or responsib* or "own care" or empower* 36538

#19 (consumer* or patient*) NEAR/2 (attitude* or knowledge* or practice or behavio*) 11978

#20 health NEAR/2 (attitude* or knowledge or practice* or behavio*) 25969

#21 sedentary NEXT behavio* 2391

#22 lifestyle* or life NEXT style* 23503

#23 personalization or personalisation or medtech or healthtech or "med tech" or "health tech" 403

#24 (personalised or personalized) NEAR/2 (health or medic* or wellness or wellbeing) 1564

#25 #14 or #15 or #16 or #17 or #18 or #19 or #20 or #21 or #22 or #23 or #24 111599

#26 #13 and #25 984

#27 MeSH descriptor: [Europe] explode all trees 29047

#28 (Europe* or andorra or Austria* or belgium or Albania* or Estonia* or Latvia* or Lithuania* or "bosnia and herzegovina" or Bosnian or Bulgaria* or Croatia* or "czech republic" or hungary or Hungarian or kosovo or "republic of north macedonia" or moldova or montenegro or poland or polish or "republic of belarus" or Romania* or Serbia* or Slovakia* or Slovenia* or ukraine or france or French or german* or gibraltar or “united kingdom” or

“channel islands” or england or English or ireland or irish or scotland or Scottish or wales or welch or Britain or british or greece or italy or Italian or liechtenstein or luxembourg or cyprus or malta or sicily or monaco or netherlands or dutch or portugal or portuguese or "san marino" or denmark or Danish or Iceland* or norway or Norwegian or sweden or Swedish or spain or spanish or switzerland or swiss or Armenia* or azerbaijan or Georgia* or ussr or Russia* or kazakhstan or kyrgyzstan or Uzbekistan or balkan):TI,AB 119112

#29 MeSH descriptor: [United States] explode all trees 19262

#30 ("United States" or USA or "North America" or "North American"):TI,AB 33849

#31 (“united states” or alabama or georgia or kentucky or maryland or “new York” or “north Carolina” or ohio or pennsylvania or “south Carolina” or tennessee or virginia or “west virginia” or illinois or indiana or michigan or minnesota or wisconsin or delaware or "district of columbia" or iowa or kansas or missouri or nebraska or “north Dakota” or oklahoma or “south Dakota” or connecticut or maine or massachusetts or “new Hampshire” or “rhode island” or vermont or idaho or montana or oregon or washington or wyoming or alaska or california or hawaii or arkansas or florida or louisiana or mississippi or arizona or colorado or nevada or “new Mexico” or texas or utah):TI,AB 46828

#32 #27 or #28 or #29 or #30 or #31 198783

#33 #26 and #32 221

OpenGrey

Search Strategy:

wearables OR “fitness trackers” OR “smart glasses” OR “smart watch*” OR “google watch*” OR wristband* OR “apple watch*” OR “microsoft watch*” OR fitbit* OR “Omron heart guide” OR “samsung gear 2” OR “get active slim” OR “jawbone UP” OR “Google Glass” OR GoQii OR HoloLens OR garmin OR “wearable tech*” OR “wearable ecg” OR smartwatch* (18)

Google Scholar

Search Strategy:

(wearable OR “fitness trackers” OR “smart glasses” OR “smart watch*” OR “google watch*” OR wristband* OR “apple watch*” OR “microsoft watch*” OR fitbit*) AND (self-manage* OR "self care" OR responsib* OR "own care" OR empower*) (2120)

Up to and including page 15 of the results (150)

The Health Foundation and The King’s Fund

Search Strategy (keyword terms were searched separately, as the sites did not support the use of Boolean operators):

wearables	“microsoft watch*”	GoQii
“fitness trackers”	fitbit*	HoloLens
“smart glasses”	“Omron heart guide”	Garmin
“smart watch*”	“samsung gear 2”	“wearable tech*”
“google watch*”	“get active slim”	“wearable ecg”
wristband*	“jawbone UP”	smartwatch*
“apple watch*”	“Google Glass”	

The Nuffield Trust

Search Strategy:

wearables OR "fitness trackers" OR "smart glasses" OR "smart watch*" OR "google watch*" OR wristband* OR "apple watch*" OR "microsoft watch*" OR fitbit* OR "Omron heart guide" OR "samsung gear 2" OR "get active slim" OR "jawbone UP" OR "Google Glass" OR GoQii OR HoloLens OR garmin or "wearable tech*" OR "wearable ecg" OR smartwatch* (4422)

Publication Limits: Research Report, Journal Article, Book (525)
