# Supplementary material for: Wearing the Future—Wearables to Empower Users to Take Greater Responsibility for Their Health and Care: Scoping Review
Source: JMIR Mhealth Uhealth. 2022 Jul 13;10(7):e35684. doi: 10.2196/35684 (PMC9330198; doi:10.2196/35684)
Supplement: Multimedia Appendix 2 [file mhealth_v10i7e35684_app2.pdf]

Data Charting Date:	
**Scoping Review Details**	
Title	
Research Question	
Aims	
Methodology (Quantitative/Qualitative/Mixed)	
Selection Criteria	
Relevant Findings	
Summary	
Wearable (Brand/Model)	
Strengths & Limitations	
**Source Details**	
Author(s)	
Date	
Journal	
Volume Number	
Issue Number	
Page Numbers	
Country	
**Other Potential Sources**	
