# Supplementary material for: Wearing the Future—Wearables to Empower Users to Take Greater Responsibility for Their Health and Care: Scoping Review
Source: JMIR Mhealth Uhealth. 2022 Jul 13;10(7):e35684. doi: 10.2196/35684 (PMC9330198; doi:10.2196/35684)
Supplement: Multimedia Appendix 3 [file mhealth_v10i7e35684_app3.pdf]

First Author	Year	Location	Study Design	Summary of Key Findings	Wearable “Brand (Model)”
**Auerswald** [65]	2020	Europe Germany	Pilot and Feasibility	Activity trackers had a high acceptance rate and significantly increased average step counts. Increased usage was associated with significantly more daily steps.	Fitbit (Zip)
**Belsi** [66]	2016	UK	Focus Groups	Perceived benefits of wearables to patients: more control over their condition; greater awareness of progress; empowering self-management; and improved communication with clinicians.	Prototype Device
**Coughlin** [67]	2020	USA	Review	Potential feasibility and preliminary efficacy of the use of consumer wearables to promote physical activity among breast, prostate, and colorectal cancer survivors.	ActiGraph<br>Fitbit (One, Charge, Flex, Zip)<br>Jawbone (UP2, UP24)<br>Garmin (Vivoactive, Vivofit 2, Vivosmart)<br>Polar (A300, 360)
**DiFrancisco-Donoghue** [68]	2018	USA	Randomised Controlled Trial (RCT)	Using activity trackers with weekly challenges more effectively increased activity relative to activity trackers alone, especially for overweight medical students.	Fitbit (Flex)
**Friel** [69]	2020	USA	Observational	Activity tracker users, characterised by more autonomous motives, presented with higher moderate to vigorous physical activity (MVPA).	Any<br>Most common: Fitbit
**Goode** [70]	2017	USA	Systematic Review	From 12 trials examining accelerometer interventions on physical activity, there was a small significant effect for increasing physical activity; intervention duration was the only moderator found to significantly explain the high heterogeneity.<br>From 11 trials examining the effects of accelerometer interventions on weight, there was a small significant effect for weight loss without significant moderators.	Activity Coach<br>Aipermon (Aipermotion 440)<br>BioTrainer<br>BodyMedia SenseWear Armband<br>DirectLife<br>Fitbit<br>GRUVE triaxial<br>Lifecorder PlusVR triaxial<br>PAM (AM101)<br>Polar FA20<br>SenseWear Pro Armband<br>Suzuken-Kenz (Lifecorder)<br>Uniaxial accelerometer
**Henriksen** [71]	2020	Europe Norway	Mixed-Method	Motivations for wearing the activity tracker were that they were: asked to; and able to track progress in their physical activity.<br>Perceived usefulness included: time-keeping; pulse and sleep tracking; becoming more conscious about daily activity; and improved understanding of which activity types were more effective for energy expenditure.<br>Measurement inaccuracies, and limited instruction for use, led to annoyance. The device seemed large, unattractive, and complicated.	Polar (M430 AT)
**Janevic** [72]	2020	USA	Randomised Controlled Pilot and Feasibility Trial	Over 90% of participants rated trackers as easy to use, but some had technical or dexterity-related difficulties. Tracker usage was not associated with greater improvement in functioning or walking.	Fitbit (Zip)

First Author	Year	Location	Study Design	Summary of Key Findings	Wearable "Brand (Model)"
**Kim** [73]	2018	USA	Cluster RCT	The control group showed a significant reduction in MVPA. The intervention group (using an activity tracker) showed no changes in MVPA, increased sedentary time and decreased time spent in light intensity activity. Hence, the study found null effects of using the tracker to promote activity.	Misfit (Flash)
**Lewis** [74]	2020	USA	Pilot RCT	Pedometers or activity trackers can improve health, with a small-to-medium effect of the activity tracker on physical activity and health compared to a pedometer.	SenseWear
**Lugones-Sanchez** [75]	2020	Europe Spain	RCT	The smartphone and smartband intervention group showed decreased body fat percentage and body mass index, and even increased weight loss relative to standard counselling at three months.	Xiaomi (Mi Band 2)
**Papi** [76]	2015	UK	Focus Groups	Appearance and comfort were key in influencing user acceptance of wearables. Patients supported the use of wearables during rehabilitation, recognising the associated benefits in terms of: monitoring progress; adhering to exercise; and informing interactions with clinicians.	Prototype Device
**Rieder** [77]	2021	Europe Switzerland	Interviews	Wearables can have positive and negative effects on perceptions of users' own self-efficacy. Such perceptions can be affected by internal and external contextual factors (certain of which may compound or neutralise the impact on self-efficacy), and in turn affect users' compliance.	Focus: wristbands/watches (Apple, Fitbit, Garmin, Xiaomi)
**Rodgers** [78]	2019	USA	Review	Numerous challenges may hinder the adoption of wearables. Widespread adoption for rehabilitation may rely on applying an understanding of end-usage to development of the technology. Routine, long-term usage introduces challenges not addressed in shorter studies (including, durability and usability).	In general
**Rupp** [79]	2018	USA	Longitudinal	Self-efficacy, activity and personality traits, indirectly increased the desire to use fitness wearables, and influenced the saliency of perceived motivational affordances. Such affordances, trust and usability, were associated with greater intent to use fitness devices.	Basis (Peak)<br>Fitbit (Flex, Charge-HR, Surge, Zip)<br>Microsoft (Band)
**Shin** [80]	2019	USA	Systematic Review	Emerging themes related to: technology and data privacy; patient treatment and self-monitoring; behaviour change; and acceptance and adoption. Research is required to understanding the rich human-information interaction that is enabled by the adoption of wearables.	Actical<br>ActiGraph<br>Basis<br>BodyMedia (FIT)<br>DirectLife<br>Fitbit (Flex, One, Zip)<br>Jawbone (UP)<br>Lumoback<br>Misfit (Shine)<br>Nike+ (Fuelband)<br>Withings (Pulse)

First Author	Year	Location	Study Design	Summary of Key Findings	Wearable "Brand (Model)"
**Soliño-Fernandez** [81]	2019	USA	Cross-Sectional	Despite concerns about economic benefits, privacy and accuracy, approximately 70% of respondents expressed a willingness to adopt health-insurance use-cases based on wearables. This willingness was greater among consumers in use-cases involving health promotion and disease prevention. Financial incentives effectively boosted the willingness in consumer use-cases involving participation for: health promotion; personalised products and services; and renewal discounts.	In general
**Stiglbauer** [82]	2019	Europe Austria	Longitudinal RCT	The fitness tracker had a small but positive effect on users' perceived health and accomplishment. Health consciousness increased for all participants. The positive effects on indicators of health and wellbeing were more pronounced when the companion app was used.	Xiaomi (Mi Band 2)
**The Nuffield Trust** [83]	2016	UK	Report (Literature Review, Interviews, Case Studies)	Monitoring with wearables can improve diet, exercise and medication adherence, but sustaining engagement is a challenge with some studies reporting negative findings. Most of the evidence relates to professionally recommended devices which is known to benefit adherence, and potentially have very positive results on outcomes and resourcing when data is sent to healthcare professionals.	In general
**Wulfovich** [84]	2019	USA	Mixed-Method	Wearables can facilitate self-management and improve wellbeing. To improve self-efficacy, wearables should be more: user-friendly; battery-efficient; accurate; and personalised, being context-aware based on user's routine, momentary state and healthcare needs, to provide timely, non-judgmental feedback.	Apple (Watch)<br>Fitbit<br>Garmin<br>Jawbone (UP)<br>Nike+<br>Samsung (Gear)
