# Supplementary material for: Wearing the Future—Wearables to Empower Users to Take Greater Responsibility for Their Health and Care: Scoping Review
Source: JMIR Mhealth Uhealth. 2022 Jul 13;10(7):e35684. doi: 10.2196/35684 (PMC9330198; doi:10.2196/35684)
Supplement: Multimedia Appendix 4 [file mhealth_v10i7e35684_app4.pdf]

Study Characteristics	Number of studies (out of 20)
**Year**	
2015	1
2016	2
2017	1
2018	3
2019	5
2020	7
2021	1
**Country**	
UK	3
USA	12
Other European Countries (Austria, Germany, Norway, Spain, and Switzerland)	5
**Study Design**	
Quantitative	10
Qualitative	8
Mixed-Methods	2
**Funding Sources Declared**	
Yes	13
No	7
