# Supplementary material for: Wearing the Future—Wearables to Empower Users to Take Greater Responsibility for Their Health and Care: Scoping Review
Source: JMIR Mhealth Uhealth. 2022 Jul 13;10(7):e35684. doi: 10.2196/35684 (PMC9330198; doi:10.2196/35684)
Supplement: Multimedia Appendix 5 [file mhealth_v10i7e35684_app5.pdf]

First Author	Funding Details
**Auerswald** [65]	Project "Prevention in Stationary Care" (AOK PLUS—Health insurance Company for Saxony and Thuringia, Germany) and by an ESF (European Social Fund) doctoral scholarship (Sächsische Aufbaubank-Förderbank, SAB) to T.A.
**Belsi** [66]	Supported by the Medical Engineering Solutions in Osteoarthritis Centre of Excellence, funded by the Wellcome Trust and the EPSRC (088844/Z/09/Z). Researchers' decisions have been entirely independent from funders.
**DiFrancisco-Donoghue** [68]	In-house New York Institute of Technology College of Osteopathic Medicine grant.
**Goode** [70]	Based on research conducted by the Evidence-based Synthesis Program (ESP) Center located at the Durham VA Medical Center, Durham, NC, funded by the Department of Veterans Affairs, Veterans Health Administration, Office of Research and Development, Quality Enhancement Research Initiative.
**Henriksen** [71]	UiT The Arctic University of Norway's thematic priority grant "Personalized medicine for public health" funded the PhD-grant for the first author. UiT also funded scientific positions for all co-authors. Tromsø County (grant TFK 2016–058) funded data collection and activity trackers in the feasibility study, as well as qualitative data collection and transcription of interviews. The University Hospital North Norway and Stamina Helse contributed to study planning and implementation of the feasibility study. The MILLS fund "1 kr til hjertesaken" contributed funding for the second round of interviews.
**Janevic** [72]	Grant from the National Institutes of Health (P30 AG015281) and the Michigan Center for Urban African American Aging Research, by grants from the National Institute on Aging (K01 AG050706-01A1 to MRJ), and by the UM OAIC Pepper Center 2017 Pilot Grant (Janevic, PI). John Piette is a VA Senior Research Career Scientist and is funded by the Michigan Center for Diabetes Translational Research (NIH Grant P30DK092926).
**Kim** [73]	Early Career Investigator Research Grant from the Society of Health and Physical Educators in America (SHAPE-America).
**Lewis** [74]	Funded by the American Heart Association (16PRE27090012) and the APC was funded by California State Polytechnic University, Pomona.
**Lugones-Sanchez** [75]	Funded by the Spanish Ministry of Science and Innovation, Instituto de Salud Carlos III, and co-funded by the European Union (ERDF/ESF, "Investing in your future") (RD12/0005/0001, RD16/0007/0003, RD16/0007/0005, RD16/0007/0008, RD16/0007/0009 and PI16/00101, PI16/00952, PI16/00765, PI16/00659, PI16/00421, PI16/00170, FI17/00040). Gerencia Regional de Salud de Castilla y Leon (GRS 1277/B/16) also collaborated in the funding of this study. They played no role in the study design, data analysis, reporting results, or decision to submit the manuscript for publication.
**Papi** [76]	Supported by the Medical Engineering Solutions in Osteoarthritis Centre of Excellence, funded by the Wellcome Trust and the EPSRC (088844/Z/09/Z).
**Rodgers** [78]	No financial support.
**Rupp** [79]	The Learning Institute for Elders (LIFE) at the University of Central Florida (UCF) under the Richard Tucker Applied Gerontology Grant.
**Soliño-Fernandez** [81]	No financial support.
