# Supplementary material for: Wearing the Future—Wearables to Empower Users to Take Greater Responsibility for Their Health and Care: Scoping Review
Source: JMIR Mhealth Uhealth. 2022 Jul 13;10(7):e35684. doi: 10.2196/35684 (PMC9330198; doi:10.2196/35684)
Supplement: Multimedia Appendix 6 [file mhealth_v10i7e35684_app6.pdf]

First Author	Health Care Providers—Benefits & Involvement	Behavior Change	Barriers to Use
**Auerswald** [65]	✓	×	✓
**Belsi** [66]	✓	✓	×
**Coughlin** [67]	×	×	✓
**DiFrancisco-Donoghue** [68]	✓	×	✓
**Friel** [69]	×	✓	×
**Goode** [70]	✓	✓	×
**Henriksen** [71]	×	✓	✓
**Janevic** [72]	✓	✓	✓
**Kim** [73]	×	✓	✓
**Lewis** [74]	×	✓	×
**Lugones-Sanchez** [75]	✓	✓	×
**Papi** [76]	✓	×	✓
**Rieder** [77]	×	✓	✓
**Rodgers** [78]	×	×	✓
**Rupp** [79]	×	✓	✓
**Shin** [80]	✓	✓	✓
**Soliño-Fernandez** [81]	×	✓	×
**Stiglbauer** [82]	×	✓	×
**The Nuffield Trust** [83]	✓	✓	×
**Wulfovich** [84]	✓	✓	✓
